# Circulating microRNAs in Alzheimer’s disease: the search for novel biomarkers

**DOI:** 10.3389/fnmol.2013.00024

**Published:** 2013-08-30

**Authors:** Véronique Dorval, Peter T. Nelson, Sébastien S. Hébert

**Affiliations:** ^1^Axe Neurosciences, Centre de Recherche du Centre Hospitalier Universitaire de Québec (Centre Hospitalier de l’Université Laval), QuébecQC, Canada; ^2^Département de Psychiatrie et de Neurosciences, Université Laval, QuébecQC, Canada; ^3^Sanders-Brown Center on Aging, University of Kentucky, LexingtonKY, USA

**Keywords:** microRNA, Alzheimer’s disease, biomarker, diagnosis, mild cognitive impairment

## Abstract

Alzheimer’s disease (AD) is the most common neurodegenerative disease in the elderly. While advancements have been made in understanding the genetic and molecular basis of AD, the clinical diagnosis of AD remains difficult, and post-mortem confirmation is often required. Furthermore, the onset of neurodegeneration precedes clinical symptoms by approximately a decade. Consequently, there is a crucial need for an early and accurate diagnosis of AD, which can potentially lead to strategies that can slow down or stop the progression of neurodegeneration and dementia. Recent advances in the non-coding RNA field have shown that microRNAs (miRNAs) can function as powerful biomarkers in human diseases. Studies are emerging suggesting that circulating miRNAs in the cerebrospinal fluid and blood serum have characteristic changes in AD patients. Whether miRNAs can be used in AD diagnosis, alone or in combination with other AD biomarkers (e.g., amyloid and tau), warrants further investigation.

## INTRODUCTION

Alzheimer’s disease (AD) is a prevalent, devastating, and progressive neurodegenerative disorder. Epidemiological studies predict that over 35 million people worldwide will be affected by 2050, thus significantly increasing social and economical burdens. There is no cure at hand, and only a few medications aimed at slowing down memory deficits and clinical symptoms are available, with limited benefits. Consequently, there is an urgent need for the identification of biomarkers that will allow the detection of AD at early (prodromal) stages, potentially leading to novel diagnostic or therapeutic strategies.

Pathologically, AD is characterized by the gradual, widespread loss of neurons, synapses, and neuropil, culminating in ~40% loss of brain mass in end-stage disease (West et al., 1994; Gomez-Isla et al., 1996). There are two main AD pathological hallmarks: extracellular amyloid (senile) plaques and intracellular neurofibrillary tangles (NFTs; Hyman et al., 2012). The amyloid plaques comprise aggregated amyloid-beta (Aβ) peptides that are generated by sequential cleavage of amyloid precursor protein (APP) by β-secretase/BACE1 and the presenilin (PSEN)-containing γ-secretase complex (Kang et al., 1987; Wolfe, 2006). The NFTs result from the abnormal aggregation of hyperphosphorylated microtubule-associated protein, tau. The reason for tau aggregation into tangles remains under investigation, but may result from an imbalance in the delicate regulation of tau kinases and phosphatases. Whereas approximately 1–5% of AD cases can be explained genetically by mutations in APP or PSEN genes, the exact cause(s) of sporadic AD remains obscure. Most experts agree, however, that sporadic AD is caused by a combination of genes and environmental factors (multifactorial), perhaps exacerbated by oxidative stress and inflammation.

Biomarkers are used to measure or indicate the effects or progress of a disease or condition. A subtype of biomarkers relates to specific and traceable biochemical molecules or compounds found in body fluids. Detection of these substances may indicate disease states or allow correlations with the progression or the susceptibility to a disease or a given treatment. They can be measured in, for instance, saliva, sweat, breath, blood/serum, urine, and cerebrospinal fluid (CSF). The collection of these biological fluids is significantly less invasive than biopsies, an important and practical issue when studying neurodegenerative disorders like AD.

Accumulating evidence suggests that circulating biomarkers may be used in AD diagnosis, the most common being Aβ peptides (Aβ40 and Aβ42, the latter being more prone to aggregation) and tau/phospho-tau (Thr181 being one of the common phospho-epitopes). While this area of research continues to progress (Tarawneh and Holtzman, 2010; Holtzman, 2011), large variability exists in the literature, hampering or delaying their routine use in the clinic (Ingelson et al., 1999). Moreover, their potential use as prodromal AD biomarkers remains uncertain. Therefore, most experts agree that additional biomarkers are required for an accurate and early diagnosis of AD vs. other potential causes of dementia. In this review, we discuss recent studies suggesting that miRNAs could function as novel, non-invasive biomarkers in AD.

## miRNAs AS BIOMARKERS

The miRNAs are a class of small (~22 nt) non-protein-coding RNAs crucially involved in the post-transcriptional regulation of gene expression. They are important for multiple biological processes such as development, proliferation, inflammation, and apoptosis (Xu et al., 2004; Pasquinelli et al., 2005; Thounaojam et al., 2013). The biogenesis and role(s) of the miRNA pathway have been recently and thoroughly reviewed by Treiber et al. (2012, and references therein). In brief, miRNAs function by binding with partial complementarity to messenger RNA (mRNA) sequences, mainly in the 3′ untranslated region (3′UTR). This targeting leads to either degradation or translational repression of the mRNA template(s), causing an overall downregulation in protein output. The miRNAs can target several disease-related genes involved in neurodegeneration (Delay et al., 2012; Abe and Bonini, 2013).

The precise mechanism(s) involved in miRNA release from cells remain largely unknown, but may involve the ceramide-dependent secretory machinery (Kosaka et al., 2010). Alternatively, there may be a passive leakage from necrotic or apoptotic cells (Zernecke et al., 2009). In any case, these small RNAs are highly stable in body fluids such as plasma and CSF (Mraz et al., 2009), making them attractive biomarkers. There are several factors involved in modulating (distant) circulating miRNAs. These small RNAs are transported in free forms, exosomes, liposomes, or high-density lipoproteins, which protect them from degradation (Vickers et al., 2011; Hu et al., 2012; the stable packaging, processing, and functionality of miRNAs in biofluids is a fascinating and important area of research mostly beyond what is addressed in the current review). While some miRNAs are ubiquitously expressed, others are present in specific cells or tissues, including the central nervous system (CNS; Landgraf et al., 2007). Furthermore, bioinformatics studies suggest that miRNA abundance is directly correlated with mRNA target activity (Dorval et al., 2012).

Interestingly, miRNAs have been described as epigenetic contributors to age-related cognitive changes (Kosik et al., 2012). It has been suggested that dysregulation of these miRNA-dependent epigenetic functions in vulnerable brain regions may lead to cognitive impairments. Accordingly, the past few years have witnessed an explosion of papers linking miRNAs to disease states, and current research efforts establish that miRNA expression profiles are altered in a variety of pathogenic conditions. This is particularly recognized in the cancer field (Sayed and Abdellatif, 2011). Interestingly, the various changes in miRNA levels are observable not only in cells/tissues directly related to disease (e.g., tumors vs. adjacent tissues), but often in the periphery or distant biological systems (e.g., tumors vs. blood). It is noteworthy that most peripheral miRNAs are also found in the brain, albeit at various levels (Hebert et al., 2013).

## CIRCULATING miRNA BIOMARKERS IN AD

### CEREBROSPINAL FLUID

Cerebrospinal fluid is a clear fluid that flows within the ventricles and around the surface of the brain and spinal cord. One primary function of CSF is to circulate nutrients within the CNS and, in turn, to act as a waste remover. The CSF is an attractive source of biomarkers as it is in direct and constant contact with the extracellular space of the brain, and can reflect biochemical and/or physiological changes that occur inside the brain.

In a pioneer study by Cogswell et al. (2008), the group performed a large-scale expression analysis of miRNAs in control and AD CSF. About 201 (out of 242 tested) miRNAs were detected above background levels, as measured by quantitative reverse transcriptase-polymerase chain reaction (qRT-PCR) using TaqMan probes (Applied Biosystems). They identified 60 miRNAs, including let-7i, that were significantly altered in AD CSF (Braak V stage) when compared to healthy elderly controls (Braak I stage; *n* = 10 per group, *P* < 0.05). Using biological pathway enrichment algorithms, the group observed an association between misregulated miRNAs and the immune system, including pathways such as *innate immunity* (e.g., miR-146b) and *T cell activation and differentiation* (e.g., miR-181a, miR-142-5p). Putative targets for these miRNAs include IRAK1, TRAF6 (Lindsay, 2008), and Bcl-2 family members (Ouyang et al., 2012). The authors suggested that abnormally expressed miRNAs in the CSF were likely derived from immune cells. This was the first study demonstrating that miRNAs can be detected in the CSF (even when initially frozen) and are altered in neurodegenerative disease conditions.

van Harten et al. (2011) confirmed that it was technically feasible to perform genome-wide expression analyses of circulating miRNAs in control and AD CSF. The authors used two stem-loop qRT-PCR methods, including: (1) an individual miRNA TaqMan qRT-PCR and (2) a Megaplex modified microarray. Using this latter approach, the authors detected 667 miRNAs from one control and one AD subject (note that more than 2,000 human miRNAs are currently registered in the miRNA database – www.mirbase.org). The authors specifically quantified and validated changes in neuronal miR-802, a suppressor of caveolin-1 (Lin et al., 2011), in the CSF of control (*n* = 8) and AD (*n* = 14) patients. Clinical tests, combined with Aβ42, t-tau, and p-tau-181 measurements in the CSF were globally consistent with the diagnosis of either group. Whether other miRNAs were misregulated in AD conditions was not evaluated.

Only recently have two critical questions been addressed in relation to CSF miRNAs in neurodegenerative diseases. The first natural question relates to *why* miRNAs are stably present in this biofluid. After all, RNAs are notoriously unstable in solution, and yet there presence has been reliably affirmed. Thus, there is a tantalizing possibility that the miRNAs in solution – and in biochemical packaging as described above – may be playing a role in the CNS. In an elegant study, Lehmann et al. (2012) demonstrated that circulating miRNAs, and in particular let-7b, could exacerbate brain damage and neurodegeneration by binding directly to the Toll-like receptor 7 (TLR7). As measured by miRNA qRT-PCR, AD CSF (*n* = 13) contained significantly higher levels of let-7b when compared to controls (*n* = 11). Here, AD patients were selected, in part, on the basis of Aβ42 and t-tau levels. Unfortunately, no correlation between these AD markers and let-7b levels was provided. However, this study demonstrates that miRNAs in CNS are bioactive, and may have paracrine/hormonal-like functions, which, if generally true, provides a novel and potentially incredibly important context for miRNA function (and pathological impact) in the brain.

A second key question is more practical, and was addressed by Alexandrov et al. (2012): is there a correlation between Aβ peptides and miRNA levels in the CSF? In this study, the patient groups consisted of six AD and six age-matched controls. Consistent with previous studies using enzyme-linked immunosorbent assay (ELISA), they reported a decrease in Aβ40 and Aβ42 in AD CSF, although this observation did not reach statistical significance (*P* ~ 0.06). Interestingly, the authors measured higher (greater than 100-fold) levels of total miRNAs (total mass) when compared to Aβ peptides, and this, both in control and AD CSF. Fluorescence-based miRNA microarrays indicated that the pro-inflammatory miRNAs miR-9, miR-125b, miR-146a, and miR-155 were significantly increased in AD CSF. These observations were further validated by a highly sensitive light-emitting diode (LED)-based Northern dot-blot analysis. This increase of specific miRNAs was extended to *in vitro* paradigms, where primary human neuronal/glial cells treated with AD-derived extracellular fluid lead to an increase of the same set of miRNAs. Significant negative correlations were observed between Aβ42 peptides and miR-137 (*r* = -0.75, *P* = 0.003), miR-181c (*r* = –0.57, *P* = 0.037), miR-9 (*r* = –0.7, *P* = 0.007), miR-29a (*r* = –0.64, *P* = 0.01), and miR-29b-1 (*r* = –0.569, *P* = 0.037), and this, in both control and AD patients. Based on these observations, it is tempting to speculate that miRs, alone or in combination with known AD biomarkers, could provide a better assessment of AD diagnosis.

### BLOOD

Blood circulates in the principal vascular system, composed of arteries and veins, to carry oxygen to and carbon dioxide from tissues. The combination of lymphocytes, monocytes, and macrophages composes the peripheral blood mononuclear cells (PBMCs) population. These blood cells are critical components in the immune system.

Schipper (2007) assessed miRNA levels in blood mononuclear cells (BMCs) derived from sporadic AD and age-matched controls (*n* = 16 per group), using a microarray chip containing 462 human miRNAs. Several miRNAs were identified to be significantly altered in AD BMCs. A large number of miRNAs, including miR-34a, miR-181b, and let-7f, were validated by miRNA qRT-PCR. Interestingly, miR-34 targets include p53 (He et al., 2007), Notch (Bu et al., 2013), and Bcl-2 (Cole et al., 2008). The let-7 targets the oncogene Ras protein, thus promoting tumorigenesis (Johnson et al., 2005). Inversely, let-7 expression is regulated by the oncogenic Myc protein (Chang et al., 2008), suggesting a regulatory feedback loop. Together, these observations highlight the importance of these miRNAs in cell/tissue homeostasis.

Geekiyanage and Chan (2011) showed by miRNA qRT-PCR a decrease in miR-137, miR-181c, miR-9, and miR-29a/b levels in the neocortical region of controls (*n* = 7) and AD subjects (*n* = 7), which negatively correlated with Aβ42 levels in post-mortem brain tissues. In a follow-up study, using the same technical approach, the group reported that the same miRNAs were also present in the blood, albeit at lower basal levels (Geekiyanage et al., 2012). They were found to be downregulated in the blood serum of mild cognitive impairment (MCI; *n* = 7) and “probable” AD patients (*n* = 7) when compared controls (*n* = 7).

Villa et al. (2013) provided further evidence that dysregulation of peripheral miRNAs might contribute to AD development. In isolated PBMCs, they first showed that the transcription factor Sp1 was regulated at a post-transcriptional level by miR-29b. Interestingly, Sp1 regulates the expression of AD-related genes such as APP (La Fauci et al., 1989) and tau (Heicklen-Klein and Ginzburg, 2000). In a cohort of 393 AD patients and 412 healthy controls, the group observed an inverse relationship between Sp1 mRNA and miR-29b levels in PBMCs (*p* = 0.002). To our knowledge, this is the first report suggesting that changes in miRNA levels (e.g., miR-29b) and its/their target(s) (e.g., Sp1) may serve as cooperative biomarkers for AD diagnosis. Whether a genuine interaction between both molecules occurs in the blood remains to be validated.

Very recently, Bekris et al. (2013) reported in an elegant 3-phase study including post-mortem brain arrays and qRT-PCR validation that plasma miR-15a correlated with neuritic plaque score and Braak stages in AD. This particular miRNA was predicted to modulate 9 AD-relevant genes, including APP (Liu et al., 2012) and tau (Hebert et al., 2010). The authors concluded that pathologically-altered brain miRNAs might be detected in CSF or plasma during life, providing further proof of principle that miRNAs are relevant clinical biomarkers of AD pathology.

## CIRCULATING miRNA BIOMARKERS IN MILD COGNITIVE IMPAIRMENT, AND CHALLENGES IN PATHOLOGICAL SPECIFICITY

Mild cognitive impairment is a term often conflated with indicating early clinical manifestation of AD, and many do indeed progress to full-blown AD clinically, although many other pathologies than AD underlie the clinical state of MCI (see below). Nevertheless, it is essential to develop tools that can accurately discriminate between normal aging, MCI, AD, and likely other cognitive disabilities. An attractive approach has recently been proposed, namely miRNA “pairs.” This concept uses, following single qRT-PCR TaqMan assays, bioinformatics to analyze the ratios of all measured miRNAs, and select the most promising pair(s) of biomarkers (Sheinerman et al., 2012). In a pilot study, 13 miRNA pairs allowed to discriminate between AD and age-matched controls, as well as between MCI and age-matched controls (*n* = 10 per group), and this, with up to 90% accuracy. The proposed sets of miRNAs could detect pre-symptomatic MCI 1–5 years before the diagnosis in 70% of cases. Finally, the same pairs of miRNAs have been able to discriminate between aged and young healthy controls (*n* = 20 per group).

There are two practical issues that are important to the clinical relevance of any biomarker: sensitivity and specificity. The issue of sensitivity is basic and relates to the fact that by the time AD is manifest as MCI, it may be too late for (at least some) therapeutic interventions. The Aβ/tau CSF studies have now shown that, as expected (Nelson et al., 2009), up to one-third of non-demented subjects harbor some AD-type pathology (Nelson et al., 2012). It is increasingly appreciated that these are the patients that should be targeted for biomarker studies as well as clinical trials.

Aspects of biomarker specificity are perhaps paramount, and often under-appreciated. Although MCI is often used to indicate an early stage of AD, MCI was originally defined according to neuropsychological features (Portet et al., 2006), which have been recognized to entail “multiple sources of heterogeneity.” As such, it is quite usual for MCI to be associated with brain pathologies other than AD: dementia with Lewy bodies (DLB), vascular pathologies, hippocampal sclerosis (HS-Aging), frontotemporal lobar dementia (FTLD), and other conditions may cause or contribute to MCI, as to dementia (Jicha et al., 2006). This highlights an important aspect of AD-related biomarkers: they are not only used in predicting whom will become demented, but also for specifying which subtype of dementia will be predominant; the importance of this specificity for clinical trials is obvious. Novel insights, relevant to this consideration, were obtained by deep sequencing miRNAs from brains of individuals with multiple different pathological diseases (AD, DLB, FTLD, and HS-Aging; Hebert et al., 2013). Although not a biomarker study *per se*, this showed that some miRNAs (particularly miR-132-5p) are downregulated in neurodegenerative diseases non-specifically. In the future, it is hoped that more specific miRNA “fingerprints” may help to distinguish the individual subtypes of neurodegenerative diseases before their earliest manifestations.

## CONCLUSION AND PERSPECTIVES

To date, most researchers have relied on the combination of Aβ peptides, total-tau, and phospho-tau (Thr181) ratios to provide the best discriminative values for individuals with or without AD. However, in most cases, large variability and differences between studied groups did not reach statistical significance, leaving inconsistencies. Without excluding the amyloid and tau biological markers, a combination of biomarkers may provide a better tool for AD diagnosis, therefore improving their clinical usefulness. Known examples include structural (e.g., hippocampal shrinkage), functional (e.g., glucose metabolism), and molecular imaging (e.g., fluorescent Pittsburgh compound B; Chintamaneni and Bhaskar, 2012).

Small non-coding RNAs, and in particular miRNAs, have come a long way in the past two decades. As discussed herein, circulating miRNAs provide an exciting and emerging research area in the biomarker field. As of now, long lists of miRNAs potentially misregulated in disease conditions have been reported, although finding overlaps is challenging (note that this is also the case for miRNA profiling studies in the brain). However, some AD-specific miRNAs were “consistently” identified, including some let-7 family members (let-7f, let-7b, and let-7i), miR-9, miR-181, and miR-29 (Maes et al., 2009). These miRNAs seem involved in processes previously associated with AD, that is to say inflammation and immunological response. Perhaps expectedly, several miRNAs and their functions as biomarkers have been patented or in the process thereof (see, e.g., www.freepatentsonline.com or www.patentlawlinks.com). Although very attractive, the applicability of miRNAs as diagnostic tools into the clinic for AD (or MCI) will require extensive validation and follow-up studies in larger cohorts of patients. This is important as AD is a heterogeneous, multifactorial disease, with often display overlapping pathologies (e.g., Aβ deposits and Lewy bodies; Gomperts et al., 2008) and/or co-morbid diagnoses (e.g., diabetes, stroke). Obviously, the ultimate goal is to provide a sensitive, reproducible, and accurate detection of AD neuropathological changes prior to the onset of the disease and the appearance of the clinical symptoms. To this end, future studies will require better neuropathological validations as well as, ultimately, far greater sample sizes for robust statistical power.

In conclusion, circulating miRNAs are amongst the promising next generation of biomarkers for AD, and ultimately the discrimination between neurodegenerative diseases. They may be small molecules, but miRNAs certainly provide a big potential for the diagnosis of human diseases.

Note: While this work was in progress, a report has been published with regards to a circulating miRNA signature in AD patients (Leidinger et al., 2013).

## Conflict of Interest Statement

The authors declare that the research was conducted in the absence of any commercial or financial relationships that could be construed as a potential conflict of interest.
