# Erratum: Circulating microRNAs in Alzheimer's disease: the search for novel biomarkers

**DOI:** 10.3389/fnmol.2013.00038

**Published:** 2013-11-11

**Authors:** Veronique Dorval

**Affiliations:** ^1^Axe Neurosciences, Centre de recherche du CHU de Québec (CHUL)Quebec, QC, Canada; ^2^Département de psychiatrie et de neurosciences, Université LavalQuebec, QC, Canada

**Keywords:** microRNA, Alzheimer's disease, biomarker, diagnosis, mild cognitive impairment

An error has been pointed out under the section CSF after our mini-review was published. At the end of the last paragraph, the reported correlations have been wrongly assigned to Alexandrov et al. The correct citation for this work is “Geekiyanage, H., and Chan, C. (2011). MicroRNA-137/181c regulates serine palmitoyltransferase and in turn amyloid beta, novel targets in sporadic Alzheimer's disease. *J. Neurosci*. 31, 14820–14830. doi: 10.1523/JNEUROSCI.3883-11.2011.” However, these results were obtained from the study of frontal cortices of Alzheimer's patients, not the CSF. Thus, caution should be taken with regards to this work, which is unfortunately no longer in the scope of the present mini-review on circulating microRNAs as biomarkers.

